# Modulation of Respiratory TLR3-Anti-Viral Response by Probiotic Microorganisms: Lessons Learned from *Lactobacillus rhamnosus* CRL1505

**DOI:** 10.3389/fimmu.2014.00201

**Published:** 2014-05-12

**Authors:** Haruki Kitazawa, Julio Villena

**Affiliations:** ^1^Food and Feed Immunology Group, Laboratory of Animal Products Chemistry, Department of Science of Food Function and Health, Graduate School of Agricultural Science, Tohoku University, Sendai, Japan; ^2^Immunobiotics Research Group, Tucuman, Argentina; ^3^Laboratory of Immunobiotechnology, Reference Centre for Lactobacilli (CERELA-CONICET), Tucuman, Argentina

**Keywords:** *Lactobacillus rhamnosus* CRL1505, TLR3, respiratory immunity, respiratory syncytial virus, immunobiotics

## Abstract

Respiratory syncytial virus (RSV) is the leading cause of lower respiratory tract illness in infants and young children. Host immune response is implicated in both protective and immunopathological mechanisms during RSV infection. Activation of Toll-like receptor (TLR)-3 in innate immune cells by RSV can induce airway inflammation, protective immune response, and pulmonary immunopathology. A clear understanding of RSV–host interaction is important for the development of novel and effective therapeutic strategies. Several studies have centered on whether probiotic microorganisms with the capacity to stimulate the immune system (immunobiotics) might sufficiently stimulate the common mucosal immune system to improve defenses in the respiratory tract. In this regard, it was demonstrated that some orally administered immunobiotics do have the ability to stimulate respiratory immunity and increase resistance to viral infections. Moreover, during the last decade scientists have significantly advanced in the knowledge of the cellular and molecular mechanisms involved in the protective effect of immunobiotics in the respiratory tract. This review examines the most recent advances dealing with the use of immunobiotic bacteria to improve resistance against viral respiratory infections. More specifically, the article discuss the mechanisms involved in the capacity of the immunobiotic strain *Lactobacillus rhamnosus* CRL1505 to modulate the TLR3-mediated immune response in the respiratory tract and to increase the resistance to RSV infection. In addition, we review the role of interferon (IFN)-γ and interleukin (IL)-10 in the immunoregulatory effect of the CRL1505 strain that has been successfully used for reducing incidence and morbidity of viral airways infections in children.

## Introduction

The first isolation of human respiratory syncytial virus (RSV) was performed in 1955 from a captive chimpanzee. The virus was quickly identified as a major respiratory pathogen in infants and children (1). RSV is a negative-strand, non-segmented RNA pneumovirus of the family *Paramyxoviridae*, and a highly contagious virus. Significant epidemiological studies have characterized RSV to be a relevant human pathogen that causes a major health burden worldwide (World Health Organization, www.who.org).

Respiratory syncytial virus causes cold-like symptoms in most healthy adults and children. In infants and young children predisposed to respiratory illness, however, RSV infection is more likely to move into the lower respiratory tract, leading to pneumonia and bronchiolitis (2). RSV has been also identified as an important cause of morbidity and mortality in the elderly, patients with chronic obstructive pulmonary disease, and transplant patients (3).

During the past years, a great advance in the knowledge of the pathogenesis and the immune response against RSV has been achieved. RSV targets both type I alveolar and non-basilar airway epithelial cells and possibly alveolar macrophages. These changes in the respiratory mucosa results in the damage of respiratory epithelial cells and the impairment of their ciliary actions. Although RSV is not a highly cytopathic virus, peribroncheal mononuclear cell infiltration, submucosal edema, mucus secretion, and sometimes syncytia are observed in the lung of RSV-infected hosts (4). In addition, several studies demonstrated that the host immune response to RSV is implicated in both protective and immunopathological mechanisms. Although inflammation elicited in response to RSV is designed to destroy, dilute, and/or sequester the virus, it can also contribute to the injury of lung tissue as a collateral damage. Indeed, the incapacity of the host to control inflammation in RSV infection correlates with the difficulty to limit virus spread, reduce the extension of lung damage and proceed onward to a phase of resolution. It is likely that understanding the pathogenesis of RSV disease, including the immune response to infection, will help to develop novel immunoregulatory therapeutic strategies and design safe and effective vaccines.

It is clear then, that the inflammatory response to RSV is complex, and refractory to treatments with antivirals and glucocorticoids, which are the standard approaches. The immumodulatory impact of probiotic is of great interest considering that these microorganisms are able to modify the responses of mucosal tissue to subsequent pro-inflammatory challenge. Moreover, several studies have centered on whether probiotic microorganisms with the capacity to stimulate the immune system (immunobiotics) might stimulate the common mucosal immune system to improve respiratory tract defenses. In this regard, it was demonstrated that some orally administered immunobiotics do have the ability to stimulate respiratory immunity and increase resistance to viral infections. During the last decade, scientists have significantly advanced in the knowledge of the cellular and molecular mechanisms involved in the protective effect of immunobiotics in the respiratory tract.

This review examines the most recent work dealing with the use of immunobiotic strains to improve resistance against viral respiratory infections. More specifically, the article review the mechanisms involved in the capacity of the immunobiotic strain *Lactobacillus rhamnosus* CRL1505 to beneficially modulate the immune response triggered by Toll-like receptor (TLR)-3 activation in the respiratory tract and to increase the resistance to RSV infection. In addition, we will discuss the role of interferon (IFN)-γ and interleukin (IL)-10 in the immunoregulatory effect of the CRL1505 strain that has been successfully used for reducing incidence and morbidity of viral airways infections in children (5).

## Innate Immune Responses Against RSV

It is known that the initiation of the mucosal and systemic immune responses to respiratory virus requires the recognition by the immune system of pathogen-associated molecular patterns (PAMPs). Recognition of viral PAMPs is achieved by cellular receptors known as pattern recognition receptors (PRRs) that are expressed in both respiratory epithelial cells and immune cells. PRRs sensors include the TLRs; C-type lectin receptors and; RNA-sensing RIG-I-like receptors (RLRs) including melanoma differentiation-associated protein 5 (MDA5) and, retinoic acid-inducible gene I (RIG-I) (6).

Double-stranded RNA (dsRNA) is a replication intermediate of several virus that is able to sensitize innate immune system through TLR3. dsRNA is observed during most RNA virus replications like RSV. The important role of TLR3 in anti-viral immunity has been experimentally proved using TLR3 knockout mice and an artificial dsRNA, the synthetic dsRNA polyinosinic–polycytidylic acid [poly(I:C)]. TLR3-deficient mice have been found to have their anti-viral immune response impaired in challenge-experiments with dsRNA or poly(I:C) (6). Then, TLR3 is considered a major PRR against virus in animal cells. In fact, epithelial cells from the respiratory mucosa over-express TLR3 when challenged with respiratory viruses and, this overexpression of TLR3 allow cells to detect virus and acquire resistance (7, 8).

**Figure 1:**
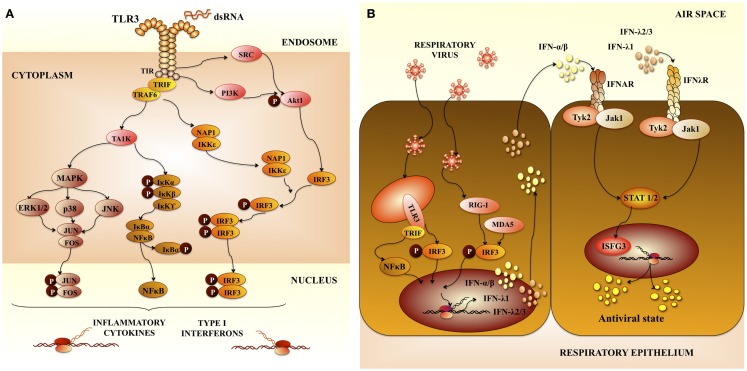
**Pattern recognition receptors in respiratory anti-viral immunity**. **(A)** Toll-like receptor 3 (TLR3) signaling pathway. TLR3 mediates signaling via the adaptor protein TRIF (TIR-containing adaptor molecule-1). The TIR domain of TRIF is essential for binding to the TIR domain of TLR3. TRIF-1 is localized in the cytoplasm of resting cells, when TLR3 is activated, TRIF co-localizes with endosomal TLR3. Then TRIF dissociates from TLR3 and co-localize with downstream-signaling molecules. The serine-threonine kinases, TANK-binding kinase 1 (TBK1) and IkB kinase-related kinase-e (IKK-e) are activated once TRIF interact with them. As a result of this activation, IRF-3 is phosphorylated. TRAF3 and NF-kB-activating kinase (NAK)-associated protein 1 (NAP1) participates in the recruitment of IRF-3 kinases and in IRF-3 activation. This pathway results in the induction of type I interferons (IFNs). In addition, mitogen-activated protein kinases and (MAPK) and NF-kB pathways are activated, which results in the induction of genes involved in inflammatory responses. **(B)** Anti-viral immune response in airway epithelial cells mediated by pattern recognition receptors and type I interferons (IFNs). Type I IFNs produced are secreted by virus-infected cells and signal in neighboring cells through the IFN-α/β receptor complex (IFNAR). This receptor is constituted by two protein subunits called IFNAR1 and IFNAR2, which are present on the surface of cells. Interaction of type I IFNs with IFNAR in neighboring cells enhance the production of type I IFNs and other inflammatory cytokines. Activation of IFNAR by IFN-α or IFN-β leads to activation of Jak1 and Tyk2 kinases, which phosphorylate the STAT transcription factors. Then, STAT heterodimers (STAT1/STAT2) or homodimers (STAT1) are generated. IRF-9 together with phosphorylated STAT1 and STAT2 form a complex called interferon-stimulated gene factor 3 (ISGF3). This complex activates the transcription of ISGs inducing an anti-viral state in the cell.

Respiratory syncytial virus predominantly infects primary airway epithelial cells, but can also infect other structural airway and immune cells. Upon viral entry and activation of signaling complexes including TLR3 (Figure 1A) (6, 9), inflammatory cytokines and chemokines are expressed and secreted in airway cells (10). In addition, respiratory epithelial cells and infiltrating leukocytes produce large amounts of anti-viral molecules, such as type I IFN. Type I IFNs signal through its receptor and induce the transcription of many interferon responsive genes (ISGs). The products of these genes limit virus replication and enhance the immune response (Figure 1B) (10).

**Figure 2:**
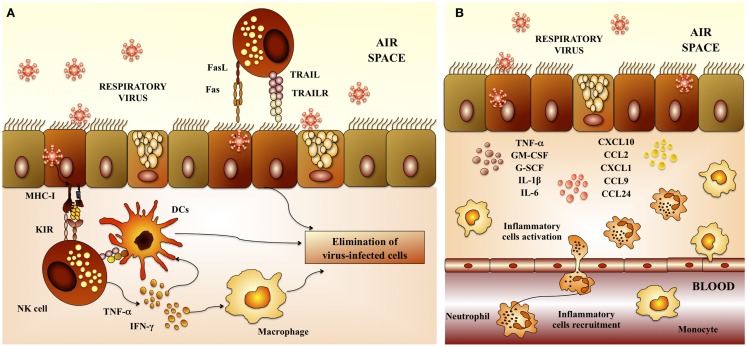
**Respiratory anti-viral innate immune response**. **(A)** Activity of natural killer cells. Natural killer (NK) cells are involved in the elimination of virus-infected cells because of their cytotoxic capacities. NK cells are recruited to the lungs early after respiratory virus infection. Dendritic cells (DCs) potentiate NK-cell activation and cytotoxicity. In addition, alveolar macrophages are also required to activate NK cells. **(B)** Inflammatory response. Epithelial cells and macrophages are crucial in the innate immune response to respiratory virus. Several chemokines and cytokines including IL-8/CXCL8, IP-10/CXCL10, MCP-1/CCL2, MIP-1a/CCL3, MIP-1b/CCL4, RANTES/CCL5, IL-6, TNF, and IL-1 are produced by epithelial cells and macrophages in response to virus infection. Upregulation of these cytokines and leads to recruitment of neutrophils, which constitute the majority of infiltrating cells. While neutrophils may mediate elimination of virus-infected cells, their high numbers, ability to secrete further cytokines and chemokines, and degranulation products may contribute to respiratory virus-induced immunopathogenesis.

Proliferation and activation of NK cells, as well as its anti-viral capacities are also important for the protection against RSV. An emerging trend born from multiple clinical studies of severely RSV-infected infants is a failure to generate a robust NK-cell response (11–13). In addition to their anti-viral activities, NK cells play a crucial role in the priming of adaptive immune responses against a variety of viral infections. Indeed, the recruitment and activation of IFN-γ-producing NK cells to the site of inflammation plays a critical role in the subsequent development of effector CD4 Th1 and cytotoxic T lymphocytes (CTLs) responses (14). This may occur indirectly through NK-cell licensing of DCs (Figure 2A). During this bidirectional cross-talk, IFN-γ released by NK cells activates DCs to produce IL-12, which in turn feeds back on the NK cell to further amplify IFN-γ secretion (14, 15). Of note, defective NK-cell function is strongly linked with the development of Th2-dominated immune responses in RSV infections (16).

In addition, recent studies demonstrated an important role for macrophages in providing an immediate pro-inflammatory response (17), and producing type I IFN (18) following RSV infection. Additionally, macrophages clear debris later in infection, and avoid further damage and inflammation (19). There is also evidence of activated granulocytes and inflammatory cytokines the airways of children and infants with severe RSV infection, being neutrophils the most abundant immune cells (Figure 2B). It is known that RSV-induced damage is produced mainly by an excessive infiltration of inflammatory cells into the airways and lung. Studies investigating the infiltration of immune cells into the lung and airways of RSV-infected children showed that neutrophils constituted the predominant population of infiltrating cells in nasal and bronchoalveolar (BAL) lavages. Moreover, neutrophils were also the most common cells found in autopsy tissues from infants infected with RSV (12, 13, 20, 21). RSV infection of the respiratory epithelium induces the secretion of pro-inflammatory mediators by epithelial cells and associated immune cells. The release of pro-inflammatory chemokines and cytokines as well as the upregulation of adhesion molecules, such as ICAM-1, induce and mediate the recruitment of leukocytes to the respiratory tract. Cytokines and chemokines, such as IL-1, IL-6, IL-8, IL-18, TNF, CCL2, CCL3, CCL5, CXCL8, and CXCL10 are significantly augmented in blood, BAL, and nasal aspirates from infants infected with RSV (12, 13, 20, 21). In particular, high levels of CXCL10 and CXCL8 that are major chemo-attractants for macrophages, neutrophils, and T cells, are hallmarks of RSV-infected infants (12, 20, 22). Furthermore, the levels of some of these molecules correlated with disease severity.

## Adaptive Immune Responses Against RSV

**Figure 3:**
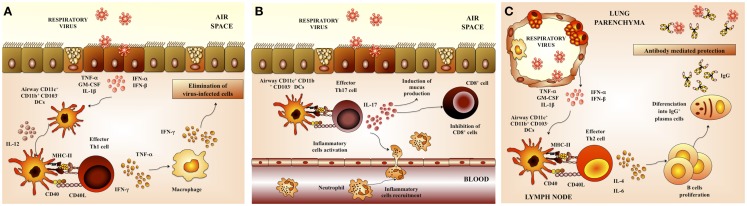
**Respiratory anti-viral adaptive immune response**. **(A)** Th1 cellular immunity. Upon respiratory virus infection of lungs, CD11b^+^ and CD103^+^ dendritic cells (DCs) are matured and migrate to the draining lymph nodes. These DCs prime Th1 cells that return to the lung and promote viral clearance. **(B)** Th17 cellular immunity. Th17 cells produce IL-17 that co-operates with IL-1β and TNF-α to induce the release of chemokines. These changes in the respiratory tract induce neutrophils recruitment and activate inflammatory responses in the lung. **(C)** Antibody-mediated immunity. Neutralizing antibodies have a critical role in protection from respiratory virus infection. Serum antibodies, mainly composed of IgG, gain access to the lungs via transduction and provide partial or complete protection against virus replication in the lungs.

Virus elimination and the recovery from primary viral respiratory infection are primarily mediated by the adaptive immune response. Both cellular and humoral immune responses act directly to eliminate viral pathogens in the respiratory tract (Figure 3).

The interaction of RSV with respiratory DCs results in activation and maturation of those cells, being both processes important in establishing virus-specific immunity. The quality and durability of the host immunity as well as the susceptibility to reinfection are significantly influenced by these early events during the initial immune response (23). Respiratory DCs that have acquired RSV antigens maturate and migrate to the lung-draining lymph nodes (LN) where they present antigens and activate antigen-specific T cells (24). In mice, lung DCs can be divided into two major populations: conventional DCs (cDCs) that are CD11c^hi^MHC-II^hi^ and plasmacytoid DCs (pDCs) that are CD11c^int^B220^+^. In addition, cDCs can be further divided into CD11b^+^CD103^−^ cDCs (CD11b^+^ cDCs) and CD11b^−^CD103^+^ cDCs (CD103^+^ cDCs) (24). Both populations of cDCs (CD11b^+^ and CD103^+^) have essentially different locations within the lung tissue. CD11b^+^ cDCs are located in the parenchyma of the lung and they promote the recruitment of leukocytes through the production of pro-inflammatory chemokines. In contrast, CD103^+^ cDCs are located in the basal lamina and they are able to extend dendrites into the airway lumen, allowing them to sample potential foreign pathogens from the airway.

After RSV challenge, the total number of lung and lung-draining LN DCs is augmented (25). However, kinetics of DCs mobilizations following acute RSV infection is different in the subepithelial CD11b^+^ cDCs when compared with parenchymal CD103^+^ DCs populations. Whereas the number of CD11b^+^ cDCs increases, the total number of CD103^+^ cDC decreases and remained low during the course of the RSV infection (26). Migrating CD11b^+^ and CD103^+^ cDCs exhibit a mature phenotype, with high expression of CD40, CD80, and CD86 molecules. Moreover, both populations exhibit a similar capacity to stimulate IFN-γ production by CD4 and CD8 T cells (26).

T cells have clear direct and indirect anti-viral effects during RSV infection. Several studies of primary and secondary RSV infections in mice models have demonstrated the central role of T lymphocytes in the pathology of RSV disease. In BALB/c mice, primary infection produces lymphocyte infiltration into the lungs and a strong production of IFN-γ by CD8 cells. Moreover, although CD4 T lymphocytes are less frequent, Th1 cells predominate even in BALB/c mice that are naturally Th2-responders (Figure 3A). CTLs appear in the lungs at day 4, peak around days 6–14, and are critical for viral clearance but can also contribute to disease (27). Other studies have strongly associated Th2 responses with increased pathology in lungs of RSV-infected mice. Decreased mucus production and lung inflammation were found in acute RSV infection when the Th2 cytokine IL-4 was depleted before viral challenge (28, 29). By contrast, decreased pulmonary pathology is associated with Th1 responses (28, 30, 31). Depletion of IL-12, a Th1 polarizing cytokine, significantly increased production of IL-13, along with increased mucus production, airway resistance, and pulmonary inflammation (30). Moreover, mice deficient in the IFN-induced transcription factor STAT1 exhibit increased production of Th2 cytokines and delayed viral clearance (31).

It has more recently been shown that Th17 cells may also play a role in effector mechanisms triggered in response to RSV. The production of IL-17 by CD4^+^ Th17 cells has both positive and negative effects in the respiratory tract. Activated Th17 cells produce IL-17 that induce the recruitment of neutrophils. Additionally, it was described that IL-17 facilitates the development tertiary lymphoid structure in infected lungs, which increase protection against RSV infection (1, 2, 32) (Figure 3B). However, IL-17 also acts synergistically with other pro-inflammatory factors and cells to exacerbate inflammatory damage and alter lung function in RSV-infected hosts. Moreover, it was recently described that IL-17 inhibits the ability of CD8^+^ cells to clear viral particles (1, 2, 32). Furthermore, IL-17 enhances IL-13 production, which promotes the activation of Th2 lymphocytes and excessive mucus production (32). As IL-17 is known to play a role in the development of asthma, its role in RSV pathogenesis was recently examined. Increased IL-6 and IL-17 levels were found in the tracheal aspirate samples from severely ill RSV-infected infants. Furthermore, IL-6, IL-17, and IL-23 were increased in RSV-infected mice, while treatment with anti-IL-17 antibodies reduced inflammation, decreased viral load, and increased antigen-specific CD8^+^ T cells in the lung (32, 33) (Figure 3B).

An effective B-cell response is also essential for resistance against viral respiratory tract infections. B cells response is reflected in the generation of antibodies capable of neutralizing the virus in both the respiratory tract and serum (Figure 3C). In this regard, a wealth of evidence indicates that mainly neutralizing antibodies confers protection against RSV infection. The F and G glycoproteins are the only viral antigens able to induce neutralizing antibodies as well as relatively long-lived protection in animal models (1). It was also reported that the prophylactic administration of RSV-neutralizing polyclonal or monoclonal antibodies is able to protect adult and infants from severe RSV disease (1, 34, 35).

## Role of TLR3 in Defense and Pathogenesis of Respiratory Virus

It is known that TLR3 has a complex role in viral infections. Challenge-infection experiments in TLR3^−/−^ animals have demonstrated that the immune response to viruses can be unaffected or impaired depending on the virus type. In fact, TLR3 has been implicated in both protective immunity and inflammatory tissue damage during viral infections. Studies of *Coxsackievirus* group B infection showed that TLR3^−/−^ mice are more vulnerable to the pathogen than wild-type mice, when considering myocarditis severity and mortality (36). In the hearts of coxsackievirus-infected TLR3-deficient mice, there was an impaired expression of IL-12p40, IL-1β, and IFN-γ, but not IFN-β, when compared with wild-type mice. On the other hand, it was reported that TLR3^−/−^ mice are more resistant to the infection with West Nile virus, indicating an important role of TLR3 in viral pathogenesis (37). It was shown that inflammatory responses and neuropathology as well as the viral load in the brain were significantly lower in TLR3^−/−^ mice compared with wild-type animals. The work clearly demonstrated that TLR3-mediated enhanced cytokine production and that this inflammatory response was critical for the alteration of the blood-brain barrier. Moreover, the magnitude of the inflammatory damage in the blood–brain barrier correlated with viral entry into the brain and the severity of lethal encephalitis.

In respiratory viral infections such as influenza virus or RSV, inflammatory response mediated by TLR3 also appears to affect the pathology induced by the virus as well as host survival.

Acute pneumonia is considered one of the most severe complications of influenza virus infection. Pneumonia develops rapidly and often results in respiratory failure and death. Remarkably, it was reported that TLR3-deficient animals are more resistant than wild-type mice to influenza virus A challenge (8). Authors described that lungs of wild-type animals presented a black hemorrhaged lung surface indicating a sever injury, whereas lungs obtained from TLR3^−/−^ knockdown mice showed only diffuse hemorrhagic foci. These results suggested that lesions induced by influenza virus A are reduced in the absence of TLR3. Lung tissue injuries correlated with a strong inflammatory response in the lungs of wild-type mice after influenza challenge, which is critically reduced in TLR3^−/−^ animals (8). Among leukocytes infiltrating the lungs of infected mice, macrophages and CD8^+^ T cells were the predominant immune cells in infected wild-type animals. However, in TLR3^−/−^ mice the number of CD8^+^ T lymphocytes was significantly lower than the one found in wild-type animals. Likewise, a significant reduction of the number of macrophages was observed in TLR3^−/−^ mice. On the contrary, neutrophils number in the lungs of TLR3^−/−^ animals was 1.5 times higher than in wild-type mice (8). Remarkably, the study showed that TLR3 deficiency caused a significant reduction of cytokine synthesis, including IL-6, IL-12p40/p70, and RANTES while other cytokines such as IFN-γ, G-CSF, IL-9, eotaxin, MCP-5, and IL-10 were increased in TLR3^−/−^ versus wild-type lungs (8). Overall, it emerges that TLR3-mediated inflammatory response would be a key point in the clinical manifestations of influenza virus-induced pneumonia.

The roles of inflammation, in general, and TLR3 in particular, in the pathogenesis of RSV have been also investigated. BALB/c mice have been used as a suitable animal model to explore the innate and adaptive immune responses to human RSV (38). The first histopathological studies of susceptible BALB/c mice challenged with human RSV were reported by Graham et al. (39). Authors demonstrated that lung injury was severe between days 5–8, resolving by day 10 after inoculation. Lung alterations were characterized by perivascular and peribronchial infiltrates of inflammatory cells. In addition, infiltration of lymphocytes and macrophages in the alveolar spaces were described. Subsequent work identified several pro-inflammatory cytokines and chemokines produced and released after RSV infection, including IP-10, KC, MIP-1α, MCP-1, RANTES, the IFN-γ regulated protein (40–42), and more recently, IL-17 (32, 33).

TLR3 can detect the dsRNA generated during the RSV replication cycle (43). It is thought that TLR3 has no or little effect on RSV clearance from the lungs. However, it is now accepted that TLR3 is necessary to regulate the respiratory immune environment. In fact, the lack of an appropriate regulation of TLR3 activation significantly contributes to the pulmonary immunopathology associated to RSV infection (44–46). It was reported that RSV-infected cells upregulate TLR3 expression and MyD88-independent chemokines, such as IP-10/CXCL10 and CCL5 after activation of the TLR3 signaling pathways by the virus (44). This increased TLR3 expression in the respiratory epithelial cells sensitizes these cells to subsequent viral dsRNA exposure and increase the production of IL-8 via NF-kB pathway (46). Moreover, it was demonstrated that RSV promotes a predominant Th1-type response when TLR3 is activated during the infection (45). By contrast, increased pathogenic Th2-biased response is generated when TLR3 is deleted, including accumulation of eosinophils in the lung and overproduction of Th2 cytokines and mucus (45).

These results are in line with the notion that the persistent unregulated inflammatory responses induced by RSV in lungs, may provide an environment that facilitates the infection with other respiratory pathogens (47). Therefore, an appropriate modulation of respiratory TLR3 could be an interesting therapeutic target not only for reducing RSV-induced lung inflammatory damage, but for avoiding subsequent infections.

## Improvement of Respiratory Anti-Viral Immunity with Immunobiotics

Certain probiotic lactic acid bacteria (LAB) strains can exert their beneficial effect on the host through their immunomodulatory activity. These strains have been termed immunobiotics (48). Although most research works concerning the immunostimulatory activities of probiotic LAB is focused on their effect in the gastrointestinal tract, several recent studies have clearly demonstrated that immunobiotics are able to improve protection against respiratory pathogens. In fact, research from the last years indicate that immunobiotic bacteria could be effectively used for the development of new prophylactic strategies that could be effective tools to protect against respiratory infections.

There are several lines of evidence that orally or nasally administered immunobiotics are capable of improving resistance against viral infections in the respiratory tract. Different aspects of respiratory anti-viral immunity can be beneficially modulated by immunobiotics, including the production of type I IFNs, the activity of NK cells, the generation of Th1 responses as well as the production of specific antibodies, and the regulation of inflammatory-mediated lung injury (Table 1).

**Table 1:** **Effect of immunobiotics on viral respiratory infections**.

Respiratory virus	Immunobiotic treatment	Protective effect	Ref.
Influenza virus H1N1	Orally administered heat-killed *B. breve* YIT4064	Reduction of accumulated symptom rate	(49)
		Improvement of survival rate	
		Improvement of serum IgG	
Influenza virus H1N1	Nasally administered heat-killed *L. casei* Shirota	Reduction of virus titer in nasal wash	(50)
		Improvement of survival rate	
		Improvement of IL-12, TNF-α, and IFN-**γ** in MLN	
Influenza virus H1N1	Orally administered heat-killed *L. casei* Shirota	Reduction of virus titer in nasal wash	(51)
		Improvement of NK-cell activity in spleen and lung	
		Improvement of TNF-α and IFN-**γ** in nasal lymphocytes	
Influenza virus H1N1	Orally administered viable *L. casei* Shirota	Reduction of virus titer in nasal wash	(52)
		Reduction of accumulated symptom rate	
		Improvement of NK-cell activity in lung	
		Improvement of IL-12 in MLN	
Influenza virus H1N1	Orally administered heat-killed *L. plantarum* L-137	Reduction of virus titer in lung	(53)
		Improvement of survival rate	
		Improvement of serum IFN-β	
Influenza virus H1N1	Orally administered lyophilized *L. gasseri* TMC0356	Reduction of virus titer in lung	(54)
		Reduction of clinical scores	
		Reduction of lung injury	
		Immune mechanism not studied	
Influenza virus H1N1	Nasally administered heat-killed *L. pentosus* S-PT84	Reduction of virus titer in BAL	(55)
		Improvement of NK-cell activity in lung	
		Improvement of IL-12 and IFN-γ in BAL	
Influenza virus H1N1	Nasally administered lyophilized *L. rhamnosu*s GG	Improvement of survival rate	(56)
		Reduction of accumulated symptom rate	
		Reduction of lung injury	
		Improvement of NK-cell activity in lung	
		Improvement of IL-1β, TNF-α, MCP-1, and IFN-γ in lung	
Influenza virus H1N1	Orally administered lyophilized *B. longum* BB536	Reduction of symptom score	(57)
		Reduction of lung injury	
		Reduction body weigh loss	
		Improvement of IL-1β, IL-6, and IFN-γ in lung	
Influenza virus H1N1	Orally administered heat-killed *L. plantarum* 06CC2	Reduction of virus titer in lung	(58)
		Reduction body weigh loss	
		Improvement of NK-cell activity in spleen	
		Improvement of INF-α, IFN-β, IFN-γ, TNF-α, IL-12, and IL-6 in BAL	
		Reduction of infiltrated neutrophils	
Influenza virus H1N1	Orally administered heat-killed *L. pentosus* b240	Improvement of survival rate	(59)
		Reduction of virus titer in lung	
		Improvement of BALF IgA and IgG	
Influenza virus H1N1	Orally administered formalin treated Lactobacilli mixture	Improvement of survival rate	(60)
		Reduction of lung injury	
		Improvement of lung IgA	
		Improvement of lung TNF-α and IL-12	
Influenza virus H1N1	Nasally administered formalin treated Lactobacilli mixture	Improvement of survival rate	(60)
		Reduction of lung injury	
		Improvement of lung IgA	
		Improvement of lung TNF-α and IL-12	
Pneumonia virus of mice	Nasally administered viable or heat-killed *L. plantarum* ATCCBAA793	Improvement of survival rate	(61)
		Reduction of virus titer in lung	
		Suppression of virus-induced CXCL10, CCL2, CXCL1, CCL9, TNF, and CCL24 in a MyD88-TLR signaling independent manner	
Pneumonia virus of mice	Nasally administered viable or heat-killed *L. plantarum* ATCC23272	Improvement of survival rate	(61)
		Reduction of virus titer in lung	
		Suppression of virus-induced CXCL10, CCL2, CXCL1, CCL9, TNF, and CCL24 in a MyD88-TLR signaling independent manner	
Poly(I:C)	Orally administered viable *L. rhamnosus* CRL1505	Reduction of lung injury	(62)
		Improvement of DCs and CD4^+^IFN-γ^+^ T cells in lung and levels of IFN-γ, IL-10, and IL-6 in BALF	
Influenza virus H1N1	Sublingual administration of lyophilized *L. rhamnosus*	Reduction of virus titer in lung	(63)
		Reduction of lung injury	
		Improvement of lung IgA, IL-12, and NK-cell activity and reduction of IL-6 and TNF-α	
Respiratory syncytial virus	Orally administered viable *L. rhamnosus* CRL1505	Reduction of virus titer in lung	(64)
		Reduction of lung injury	
		Improvement of DCs and CD4+IFN-γ^+^ T cells in lung and levels of IFN-γ, IL-10, and IL-6 in BAL	
Respiratory syncytial virus	Nasally administered viable or heat-killed *L. rhamnosus* CRL1505	Reduction of virus titer in lung	(65)
		Reduction of lung injury	
		Improvement of DCs and CD4+IFN-γ^+^ T cells in lung and levels of IFN-γ, IL-10, and IL-6 in BAL	
Influenza virus H1N1	Intragastric administration of *L. plantarum* CNRZ1997	Reduction of virus titer in lung	(66)
		Reduction of weight loss and alleviation of clinical symptoms	
		Immune mechanism not studied	
Influenza virus H1N1	Orally administered viable and non-viable *L. acidophilus* L-92	Reduction of virus titer in lung	(67)
		Improvement of NK cells activity in lungs	
		Reduction of infiltrating neutrophils	
		Increase of IL-17 in Peyer’s patches	
Influenza virus H1N1	Orally administered lyophilized *L. brevis* KB290	Alleviates clinical symptoms, loss of body weight, and the deterioration of physical conditions	(68)
		Improvement of IgA and IFN-α in BAL	
Influenza virus H1N1	Orally or nasally administered *L. plantarum* DK119	Reduction of virus titer in lung	(69)
		Reduction of body weight loss	
		Modulation of DCs and macrophages activities in lungs	
Respiratory syncytial virus – influenza virus H1N1	Orally administered viable *L. rhamnosus* CRL1505	Reduction of virus titer in lung	(70)
		Reduction of lung injury	
		Modulation of tissue factor and thrombomodulin expression in lungs	
		Improvement of IFN-γ and IL-10 in lungs	

Maeda et al. (53) showed that orally administered heat-killed *Lactobacillus plantarum* L-137 augmented the resistance against influenza virus infection by stimulating the production of type I IFN. The study showed that *L. plantarum* L-137 treatment significantly prolonged the mean survival time in mice infected with a mouse-adapted virulent strain of influenza virus H1N1, and that this effect correlated the increased production of IFN-β (53). However, detailed studies to investigate the immune mechanisms involved in *L. plantarum* L-137 activity were not performed.

Other studies emphasized the importance of IFN-γ production and NK cells activation for the protective effect of immunobiotics against influenza infection (55–57). Earlier studies with the known probiotic strain *Lactobacillus casei* Shirota clearly demonstrated the capacity of this bacterium to stimulate NK cells activity and cellular immunity in the respiratory tract. Moreover, the study showed that the Shirota strain was able to improve the resistance of mice to influenza virus challenge (50). It was found that mice receiving *L. casei* Shirota intranasally strongly induced production of IL-12 in mediastinal lymphoid nodes (MLN) cells. In addition, both IFN-γ and TNF-α were augmented in MLN cell cultures from mice receiving *L. casei* Shirota intranasally. These changes in MLN’s cytokine profile, induced by the immunobiotic treatment, explain the improvement of NK cells stimulation and the enhancement of the Th1 response (50). A second work of the same group demonstrated that orally administered *L. casei* Shirota activated the systemic and respiratory immune systems and diminished influenza virus infection severity in both aged (51) and infant mice (52). As observed in adult mice, the protective effect of the Shirota strain correlated with augmented NK-cell activity in splenocytes and lungs and enhanced IFN-γ and TNF-α production of nasal lymphocytes. More recently, it was showed that oral administration of heat-killed *L. gasseri* TMC0356 or lyophilized *L. rhamnosus* GG resulted in a higher expression of pulmonary IFN-γ and reduced pulmonary virus titers between control and lactobacilli-treated mice (54).

In an effort to evaluate the capacity of lactobacilli to reduce the pathogenesis of severe pneumovirus infection *in vivo*, Gabryszewski et al. (61), developed a model pneumonia virus of mice (PVM) infection. Authors showed that nasally administered *L. plantarum* or *Lactobacillus reuteri* were highly effective for controlling inflammation induced by PVM infection and for protecting against lethal disease. Lactobacilli treatments reduced virus recovery and diminished granulocyte recruitment, and the expression of pro-inflammatory cytokines including CXCL10, CXCL1, CCL2, and TNF (Table 1). Other studies also showed the capacity of immunobiotics to beneficially modulate the balance between pro- and anti-inflammatory mediators during respiratory virus infections. Takeda et al. (58) demonstrated that *L. plantarum* 06CC2, when orally administered, differentially modulated the production of cytokines during influenza infection. The levels of IFN-γ, IL-12, and IFN-α in infected mice administered the 06CC2 strain were significantly higher than those in the controls while the level of TNF-α was significantly lower than that in the control mice (58). Another study investigated whether that sublingual administration of *L. rhamnosus* enhanced protection against influenza virus (63). The work reported that immunobiotic treatment was able to augment T cell and NK-cell activity in the respiratory mucosa, enhancing the resistance against viral infection. Moreover, authors found that *L. rhamnosus*-treated mice had improved levels of IL-12 and reduced IL-6 and TNF-α levels in lungs when compared to controls, indicating that the probiotic treatment modulated cytokine profile in response to the infection. Taking into account that the levels of the pro-inflammatory cytokines IL-6 and TNF-α have a positive correlation with vascular dysfunction and lung inflammation, these results suggest that the reduced concentrations of some pro-inflammatory mediators would be helpful to protect against influenza virus infection (63).

The impact of immunobiotics on anti-viral humoral response has been also evaluated. Early studies from Yasui et al. (71) showed that orally administered *Bifidobacterium breve* YIT4064 augmented the production of anti-viral antibodies including anti-poliovirus, anti-influenza virus, and anti-rotavirus antibodies after the challenges with the respective viral pathogens (71). Moreover, a second work of the same group with the YIT4064 strain clearly demonstrated that the immunobiotic treatment significantly improved the protection of mice against influenza infection; and that this protective effect was related to increased anti-influenza virus IgG titers in serum (49). More recently, the ability of non-viable immunobiotics to improve respiratory anti-viral immunity was evaluated. It was reported that orally administered heat-killed lactobacilli enhanced anti-influenza antibodies in the airways. Both IgA and IgG specific antibodies significantly reduced the susceptibility of mice to influenza virus infection (59, 60). Then, immunobiotics are capable to modulate the production of systemic and mucosal antibodies against respiratory viruses (Table 1).

We aimed to evaluate whether a probiotic yogurt containing the immunobiotic strain *L. rhamnosus* CRL1505 was able to beneficially modulate both gut and non-gut related illnesses in humans. For this purpose, we performed a randomized controlled trial in children under 5 years old (62). We demonstrated that the intervention with the immunobiotic strain CRL1505 was able to reduce the frequency and severity of mucosal infections (intestinal and respiratory) in young children; and that this protective effect was related to an improvement of mucosal immunity. It was also found that in children who consumed *L. rhamnosus* CRL1505, the presence of fever and the need for antibiotic treatment were significantly reduced when compared to the placebo control group, indicating less serious infections (63). We did not study the etiology of the respiratory infections in the clinical trial, however previous epidemiological evaluations have shown that viral pathogens including RSV, human metapneumovirus, influenza A virus, parainfluenza viruses, and rhinoviruses are the major viruses responsible of respiratory tract diseases in children in our country (72). Therefore, the findings of our study suggested that administration of *L. rhamnosus* CRL1505 could be an interesting tool for reducing the incidence and severity of common childhood infectious diseases, especially those associated to viral pathogens (62).

## Distal Modulation of Respiratory Anti-Viral Immunity by *L. rhamnosus* CRL1505

Taking into consideration the results of the clinical studies, we were interested in demonstrating the capacity of *L. rhamnosus* CRL1505 to improve respiratory anti-viral immunity and to gain insight into the immunological mechanism(s) involved in the beneficial effect. Then, we evaluated the effect of the oral administration of *L. rhamnosus* CRL1505 on respiratory anti-viral immunity triggered by TLR3 activation. For this purpose, we used infant and adult BALB/c mice and the nasal administration poly(I:C) that is and artificial dsRNA analog and TLR3 ligand, to induce lung inflammation. This mice model allows us to imitate functional alterations and pro-inflammatory consequences of RNA viral infections in the lung. We showed that after nasal administration of poly(I:C) to BALB/c mice there was an increased inflammatory cell recruitment into the lung and production of pro-inflammatory cytokines, that were accompanied by a marked impairment of lung function (62) in accordance with results published by Stowell et al. (73). Increased levels of albumin concentration and lactate-dehydrogenase (LDH) activity were observed in BAL after poly(I:C) administration indicating impaired epithelial barrier function and respiratory epithelial cell death. Moreover, TLR3 activation by intranasal administration of poly(I:C) resulted in neutrophils and mononuclear cells influx into the lung (43, 62, 73).

Increased levels of respiratory MCP-1, IL-6, TNF-α, and IL-8 were observed in our *in vivo* experiments with BALB/c mice. Previous *in vitro* studies showed that stimulation of respiratory epithelial cells with poly(I:C) increases TLRs and transcription factors expression and induces the secretion of multiple cytokines and chemokines (73). Therefore, the source of pro-inflammatory cytokines and chemokines after poly(I:C) administration may be the airway epithelium. It was described that the profile of pro-inflammatory mediators induced by RSV is similar to the one triggered by poly(I:C) (43, 73), then the experimental model used in our works resembles RSV infection. Moreover, experimental RSV challenge in mice and RSV infection in children is characterized by a prominent secretion of pro-inflammatory mediators in the respiratory tract, as mentioned before. The coordinated actions of these pro-inflammatory mediators promote neutrophils and monocytes/macrophages recruitment and activation in the lung (38), also observed in our mice model (62).

Host’s inflammatory response has to be tightly regulated during acute viral lung infection. A regulated inflammatory response enables pathogen elimination without the detrimental effects of inflammation on the delicate lung tissue where gas exchange is produced. Therefore, an appropriate balance between pro-inflammatory and anti-inflammatory factors is crucial for an effective and safe response against RSV. In fact, it was described that excessive IL-10 production can induce a delayed virus clearance while exuberant production of TNF-α/IL-8/MCP-1 can lead to increased immunopathology (74). During the early stages of RSV infection, TNF-α significantly contributes to virus clearance. However, overproduction of TNF-α in the late stages of RSV infection exacerbates tissue injuries and illness (42). Interestingly, it was shown that immunopathology and lethal disease during influenza infection is prevented by IL-10 (75). IL-10 also seems to play an important role in the control of infection severity in RSV challenged hosts (75, 76). IL-10 deficiency during RSV infection did not affect lung viral titers. However, lack of IL-10 significantly increases the severity of RSV disease. Absence of IL-10 allows a greater release of inflammatory cytokines, enhanced influx of inflammatory cells, and delayed recovery (77). Then, the reduction of MCP-1, IL-8, TNF-α, and IL-6 in the lung after the challenge with poly(I:C) could explain, at least partially, the capacity of *L. rhamnosus* CRL1505 to reduce lung injuries (62). Moreover, IL-10 concentrations in the respiratory tract and serum of *L. rhamnosus* CRL1505-treated mice were significantly increased prior the challenge with poly(I:C). IL-10 would be valuable for attenuating TLR3-mediated inflammatory damage in the lungs. Consequently, *L. rhamnosus* CRL1505 treatment could be used to beneficially modulate the balance between pro- and anti-inflammatory cytokines, allowing a reduction of lung tissue damage through an effective regulation of the inflammatory response.

Oral treatment with the CRL1505 strain also increased levels of IFN-γ in the respiratory tract after poly(I:C) challenge (62). The higher levels of respiratory IFN-γ in *L. rhamnosus* CRL1505-treated mice could be related to the increased activity lung DCs that are able to augment CD3^+^CD4^+^IFN-γ^+^ T cells numbers. In addition, we found increased levels of CD11b^high^ and CD103^+^ DCs in lungs of *L. rhamnosus* CRL1505-treated mice after challenge with poly(I:C). Moreover, an improved MHC-II expression was found in both DCs populations when compared with controls. However, only CD103^+^ DCs showed higher production of IL-12 and IFN-γ in *L. rhamnosus* CRL1505-treated mice (62). In line with our results, it was reported that priming of CD4^+^DO11.10CD62L^high^ T lymphocytes with lung CD103^+^ DCs, induced CD4^+^ T cells that produce preferably IFN-γ rather than IL-4 (78).

These results of our clinical trial and the studies in mice clearly indicated that *L. rhamnosus* CRL1505 could be useful as a prophylactic agent to control viral respiratory virus since this probiotic strain is a potent inducer of anti-viral cytokines. However, further research was needed to conclusively demonstrate the protective effect of the CRL1505 strain against real viral challenges. Therefore, we next examined whether oral administration of *L. rhamnosus* CRL1505 was able to reduce the susceptibility of infant mice to RSV infection. We demonstrated that oral administration of *L. rhamnosus* CRL1505 contributed to a significant decrease of RSV titers and lung tissue damage after the challenge with the respiratory pathogen (64). The protective effect achieved by the immunobiotic strain was related to its ability to modulate the respiratory anti-viral response. As observed in poly(I:C) challenge-experiments, infant mice orally treated with the CRL1505 strain showed an early increase in the levels of respiratory TNF-α and IL-6 after RSV infection. The early increases of these cytokines together with the improved levels of IFN-γ were probably related to the higher ability of the immunobiotic bacterium to reduce viral loads. In addition, orally administered *L. rhamnosus* CRL1505 significantly augmented IL-10, which contributed to protection against inflammatory damage (64). In fact, we demonstrated that both IFN-γ and IL-10 are necessary to achieve full protection against RSV in infant mice and that these cytokines are differently involved in the immunoprotective effect of *L. rhamnosus* CRL1505. The reduction of RSV titers induced by the immunobiotic strain was abolished when blocking anti-IFN-γ antibodies were used. In addition, the reduction of lung tissue injury induced by the CRL1505 strain was partially abolished with anti-IFN-γ antibodies (64). On the contrary, the use of blocking anti-IL-10R antibodies did not affect the ability of the immunobiotic strain to reduce RSV titers. However, blocking antibodies against IL-10R significantly abolished the protective capacity of *L. rhamnosus* CRL1505 against lung tissue damage (64).

*L. rhamnosus* CRL1505 also improved lung CD103^+^MHC-II^+^ and CD11b^high^MHC-II^+^ DCs after RSV challenge (64). Considering that CD103^+^ and CD11b^high^ lung DCs are able to present RSV antigens to naïve T cells (26), and that both DCs populations are important in the generation of CD8^+^ and CD4^+^ effectors T cells, the increase of lung DCs would have a critical role in the immunoregulatory effect of *L. rhamnosus* CRL1505. It could be speculated that the immunobiotic strain would be able to improve protective adaptive immune response by beneficially modulating DCs activity, considering that activation and maturation of antigen presenting cells after RSV arrival to the lung determine the quality and durability of host immunity and influence susceptibility to reinfection (64).

Respiratory syncytial virus infection induces Th2-like inflammation in the lung. Therefore, strategies that improve Th1 responses against RSV are considered beneficial to modulate the outcome of the disease especially in young individuals. IFN-γ augments the expression of MHC-II and MCH-I in DCs and increases the cellular Th1 anti-viral immune response. These changes suppress the proliferation and activation of Th2 T cells (79). Consistent with this notion, *L. rhamnosus* CRL1505 administration to infant mice significantly increased RSV clearance and augmented respiratory IFN-γ levels. Then, modulation of respiratory immunity induced by the immunobiotic strain might contribute to an increase in Th1 response and thereby favor protective immunity against respiratory viral infections such as RSV.

**Figure 4:**
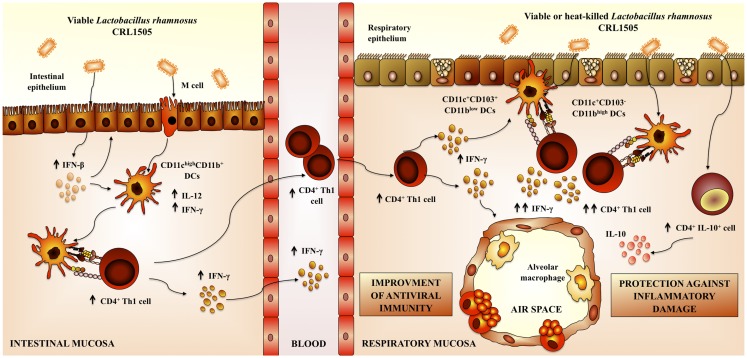
**Proposed mechanism for the immunoregulatory effect of *Lactobacillus rhamnosus* CRL1505 on respiratory anti-viral immune responses and resistance against respiratory syncytial virus**.

We were particularly interested in gaining insight into the mechanism(s) involved in the immunoprotective capacities of *L. rhamnosus* CRL1505. *In vivo* and *in vitro* experiments demonstrated that the CRL1505 strain significantly augmented the levels of IFN-γ, IFN-α, IFN-β, TNF-α, IL-10, and IL-6 in the intestine and the number of CD3^+^CD4^+^IFN-γ^+^ T cells in Peyer’s Patches. In addition, *L. rhamnosus* CRL1505 is able to improve these cytokines in blood, particularly IFN-γ, IL-10, and IL-6. The profile of blood cytokines was similar to the one in the intestinal fluid, suggesting that levels of serum cytokines are a reflection of intestinal changes (80). On the contrary, the analysis of respiratory cytokines showed that only IFN-γ, IL-10, and IL-6 were increased by *L. rhamnosus* CRL1505 (62). These same cytokines were augmented by the immunobiotic strain in serum, however, it was not possible to attribute a direct correlation between the increases in the respiratory tract and blood, because TNF-α, IFN-α, or IFN-β levels were not augmented in the airways of *L. rhamnosus* CRL1505-treated mice. Therefore, considering the ability of *L. rhamnosus* CRL1505 to augment the number of intestinal CD3^+^CD4^+^IFN-γ^+^ T cells, we hypothesized that the immunobiotic strain would induce a mobilization of these cells into the respiratory tract. We confirmed that this assumption was true after demonstrating increased numbers of CD3^+^CD4^+^IFN-γ^+^ T in the lungs mice orally treated with *L. rhamnosus* CRL1505 (62). Furthermore, the mobilization of CD3^+^CD4^+^IFN-γ^+^ T cells from the intestine to the airways and the higher production of IFN-γ could be involved in the improved anti-viral state induced by *L. rhamnosus* CRL1505 that was observed in clinical studies (5). Probably, IFN-γ secreted in response to *L. rhamnosus* CRL1505 stimulation would be capable of functionally modulate the innate immune microenvironment in the lung, inducing the activation of DCs (64) and macrophages (81). Additionally, IFN-γ would favor the generation of Th1 immunity with the consequent reduction of the damaging Th2 reactions that are associated to RSV challenge (64) (Figure 4). In addition, there is increasing information regarding the involvement of Th17 cells in respiratory virus infections such as influenza and RSV. As mentioned before, cytokines produced by Th17 cells have both positive and negative effects during RSV infections. Considering that some works have demonstrated the capacity of immunobiotics to beneficially modulate the Th17 response in respiratory allergy; it would be an interesting topic for future research to evaluate the effect of *L. rhamnosus* CRL1505 on Th17 response during RSV infection in infant mice.

## Local Modulation of Respiratory Anti-Viral Immunity by *L. rhamnosus* CRL1505

Considering that nasally administered antigens induce respiratory and systemic immune responses that are superior to those obtained with oral immunizations, we also analyzed whether the nasal administration of immunobiotics is capable of increasing resistance against poly(I:C)/RSV challenges. In addition to the CRL1505 strain, we also evaluated *L. rhamnosus* CRL1506, a strain with a strong capacity to stimulate the production of type I IFNs in intestinal epithelial cells (62, 82). Our work demonstrated that nasally administered CRL1505 or CRL1506 strains were capable of modulating TLR3-triggered anti-viral respiratory immune response, demonstrating in addition a different immunoregulatory effect for each immunobiotic strain. *L. rhamnosus* CRL1506 significantly modulated the production of type I IFN and IL-6 in the response to poly(I:C) or RSV challenges. On the other hand, priming with *L. rhamnosus* CRL1505 effectively improved levels of IFN-γ and IL-10 in the respiratory mucosa (83).

*L. rhamnosus* CRL1506 had a significant effect on epithelial cells from the respiratory tract. It is known that type I IFNs increase the expression of genes that are involved in innate anti-viral defenses and the development of a strong Th1 response. Therefore, *L. rhamnosus* CRL1506, through the stimulation of anti-viral defenses in epithelial cells, could play a significant role in the improvement of innate and specific immune responses against respiratory viral infections (83). On the other hand, nasal administration of *L. rhamnosus* CRL1505 augmented levels of BAL IFN-γ and lung CD3^+^CD4^+^IFN-γ^+^ T indicating an improvement of the respiratory Th1 response. Moreover, CRL1505 administration significantly activated CD103^+^ DCs. Those effects were not observed in mice orally treated with the CRL1506 strain. Then, *L. rhamnosus* CRL1505 would be more efficient than *L. rhamnosus* CRL1506 to stimulate CD103^+^ DCs and improve Th1 response in the lung (83). In line with this notion, recent studies suggested that respiratory CD103^+^ DCs are more potent at eliciting Th1 responses than CD11b^high^ DCs (78).

Nasal treatment with *L. rhamnosus* CRL1505 and CRL1506 significantly reduced lung injuries caused by poly(I:C). Both lactobacilli augmented IL-10 production in response to TLR3 activation, however, *L. rhamnosus* CRL1505 was more efficient than CRL1506 to increase the levels of this cytokine in the lung. Additionally, the markers of lung damage were lower in CRL1505-treated mice than in those receiving *L. rhamnosus* CRL1506 (83). Therefore, there is a direct connection between the improvement of respiratory IL-10 and the protection against poly(I:C)-induced lung damage after immunobiotic treatment. Moreover, our results indicate that CD3^+^CD4^+^IL-10^+^ T cells would be functionally and quantitatively modulated by *L. rhamnosus* CRL1505 and that these cells would be the source of the IL-10 produced after poly(I:C) challenge (83). Recently, it was reported that the majority of IL-10 produced during acute RSV infections comes from CD4^+^ T (76). Moreover, it was suggested that this cell population is involved in the protection against lung tissue alterations. Therefore, the improved numbers of lung CD3^+^CD4^+^IL-10^+^ T cells induced by nasally administered immunobiotics could have an important role in the protection against RSV challenge. It should be considered in addition that during respiratory infections, other cell populations are able to produce IL-10 (75, 76). It was described that IL-10 is produced by different CD4^+^ T cells during RSV infection. These cell populations include Foxp3^+^ regulatory T cells, IFN-γ producing Foxp3^−^CD4^+^ T cells that coproduce IL-10, and Foxp3^−^CD4^+^ T cells that do not coproduce IFN-γ (76). Moreover, it was described that a small number CD8^+^ T cells also produce IL-10 after RSV challenge (76). It would be of value to investigate whether immunobiotic treatments influence the production of IL-10 in respiratory CD4^+^Foxp3^+^, CD4^+^Foxp3^−^ IFN-γ^+^, CD4^+^Foxp3^−^IFN-γ^−^, and CD8^+^ T cells.

Nasal administration of *L. rhamnosus* CRL1505 or *L. rhamnosus* CRL1506 augmented the production of pro-inflammatory mediators and IL-10 in response to RSV infection (83). *L. rhamnosus* CRL1505 was more effective than *L. rhamnosus* CRL1506 to improve the levels of respiratory IL-10, to protect against the inflammatory damage, and to enhance virus clearance, similarly to our results using poly(I:C). This finding also supports the idea that modulation of IL-10 is an effective way to improve the outcome of RSV disease. In addition, our results demonstrated that the nasal priming with immunobiotics is an interesting alternative to achieve the immunoprotective effect in the respiratory tract; since virus titers and lung alterations were significantly lower in mice nasally treated with *L. rhamnosus* CRL1505 than in those fed with the bacteria (62, 83) (Figure 4).

Our results also demonstrated that nasally administered immunobiotics are more effective than orally delivered probiotics to improve anti-viral respiratory defenses and protect against viral infections such as RSV (62, 83).

Finally, we evaluated whether viability of the immunomodulatory lactobacilli was a necessary condition to achieve the protective effect against respiratory viral infection. Some few studies reported that nasally administered heat-killed immunobiotics are capable of improving resistance against respiratory pathogens (50, 61, 84, 85) (Table 1). In this regard, studies by Hori et al. (50) showed that the nasal priming with heat-killed *L. casei* Shirota significantly augmented the resistance of adult BALB/c mice to influenza virus by stimulating respiratory tract cellular immunity. *L. casei* Shirota strongly induced production of IL-12 in MLN cells, which stimulates cytotoxic T cells and NK cells, and enhances the Th1 response. Moreover, after influenza virus challenge, both TNF-α and IFN-γ were increased in MLN cell cultures from mice nasally treated with *L. casei* Shirota (50). In addition, it was reported an improved IFN-α production and NK activity as well as a strongly enhanced Th1 immunity in the respiratory tract of mice treated nasally with heat-killed *L. pentosus* S-PT84, which were protected against influenza virus infection (55). More recently, it was shown that nasal priming with both live and heat-killed *L. plantarum* and *L. reuteri* induces a full protection against the lethal pneumovirus infection (61). That work demonstrated that nasally administered heat-killed lactobacilli resulted in a strong regulation of virus-induced pro-inflammatory mediators and diminished virus recovery. The results of our recent experiments are in line with these previous works since administration of both heat-killed *L. rhamnosus* CRL1505 was effective to improve resistance of infant mice to RSV infection and reduce lung injuries, inducing a protective effect that was similar to the observed for the viable strain (83) (Figure 4). Interestingly, although both viable and heat-killed *L. rhamnosus* CRL1506 showed a similar capacity to reduce lung RSV titers, the viable bacteria was more effective than the heat-killed ones to reduce lung damage after RSV challenge. These differential effects achieved by viable and heat-killed lactobacilli could be explained by their specific capacities to modulate the production of IL-10 and IFN-γ during RSV infection (83). The four treatments evaluated were capable of increasing the levels of IFN-γ in the respiratory tract and decreasing viral loads. On the other hand, *L. rhamnosus* CRL1505 (viable and heat-killed) and viable *L. rhamnosus* CRL1506 but not the heat-killed CRL1506 strain reduced lung damage by increasing IL-10 concentrations. These results suggest that the immunoregulatory effect of some probiotic bacteria can be changed after heat treatment. Therefore, not all heat-killed bacteria derived from immunobiotic will maintain their immunoregulatory capacities. This fact should be considered when selecting non-viable immunobiotic strains (83).

## Conclusion

No effective therapy strategies are available at the moment for the prevention and treatment of RSV infections. Findings in RSV biology and immunopathology suggest that only the inhibition replication may not be effective for reducing lung damage during severe infection. It should be considered that once individual experiences the symptoms of RSV infection, the inflammatory response has become uncontrolled and it is not longer linked to the replication of virus directly. Then, the use of replication inhibitors to control lung damage is not useful. Immunoregulatory therapies could be more effective to control the negative sequelae of severe RSV disease.

We have demonstrated that the respiratory immune response triggered by TLR3 activation could be beneficially modulated by mucosal (oral and nasal) administration of immunobiotic lactobacilli. Moreover, those treatments are able to increase the resistance to RSV challenge in both infant and adult hosts. We also showed that the anti-viral capacities of immunobiotic lactobacilli are strain dependent, as it has been reported for other probiotic effects. Comparative studies using two *L. rhamnosus* strains of the same origin (32, 80) allow us to demonstrate that each lactobacilli strain has specific immunoregulatory effects. Each strain differentially modulates the immune response in the respiratory tract after poly(I:C) stimulation. In addition, each lactobacilli confer different degree of protection against RSV challenge and use distinct immune mechanisms (Figure 4).

Our research also demonstrated that anti-viral respiratory defenses are beneficially modulated by heat-killed immunobiotics. This implies that non-viable immunobiotics could be an interesting alternative as mucosal adjuvants to improve respiratory defenses and protect against viral infections. The use of non-viable immunobiotics or their cellular fractions could have an important impact in the prevention of viral respiratory infections in immunocompromised hosts in which the use of live bacteria might be dangerous. In addition, heat-killed immunobiotic could have several technological advantages such as easier storage, and transportation and a longer product shelf-life. Therefore, an interesting topic for future research would be the evaluation of non-viable *L. rhamnosus* CRL1505 or its cellular fractions as immunomodulators and anti-viral adjuvants in immunocompromised hosts.

## Conflict of Interest Statement

The authors declare that the research was conducted in the absence of any commercial or financial relationships that could be construed as a potential conflict of interest.
